# The Effect of Education and Awareness on the Quality-of-Life in Postmenopausal Women

**DOI:** 10.4103/0970-0218.62563

**Published:** 2010-01

**Authors:** Sedigheh Forouhari, Marjan Khajehei, Marziyeh Moattari, Mitra Mohit, Mozhgan Safari Rad, Haleh Ghaem

**Affiliations:** Fatemeh (P.B.U.H.) College of Nursing and Midwifery, Shiraz, Iran; 1 Epidemiology Department of Health and Nutrition Faculty, Shiraz University of Medical Sciences, Shiraz, Iran

**Keywords:** Education, menopause women, quality-of-life

## Abstract

**Objectives:**

Women are one of the most important parts of the family and society, and community health is dependent on provision of the needs of this group. Menopause is one of the most critical stages of life among women. One of the aims of health services for all of the people in the 21^st^ century is improvement of the quality-of-life. In menopausal women, the term quality-of-life incorporates its physical symptoms such as hot flushes, night sweats and vaginal mucosa dryness. We set out this study in order to evaluate the effect of education on the quality-of-life and the improvement of health standards in menopausal women.

**Materials and Methods:**

Sixty-two women aged 44–55 referring to and academic outpatient clinic in Shiraz were selected by simple random sampling, and allocated in two groups. Data was collected using a modified Hildich questionnaire on quality-of-life in menopause stage. Quality-of-life of the subjects (vasomotor, psychosocial, physical and sexual aspects) were evaluated prior to and 3 months after educational intervention.

**Results:**

Mean quality-of-life score in study and control groups, prior to education, was 81.7 and 74.8; changing to 75.3 and 75.8, respectively three months after intervention. The study group showed a significant improvement in their quality-of-life (*P* = 0.001). A significant difference was seen between groups in terms of changing quality-of-life after intervention (*P* = 0.001).

**Conclusions:**

Appropriate training to menopausal women improves their quality-of-life and promotes their health.

## Introduction

Women are among the most important part of any society and family, and community health provision is dependent on the fulfillment of different health needs. On the other hand, menopause is one of the most critical stages of women health.(1) Defining the age for menopause is somewhat challenging, and according to estimations from cross-sectional studies, it has been reported to average 50–52 years old.(2) Despite the rise of life expectancy, age of onset of menopause or cessation of menstruation has not changed, and women live for about 30 years (more than third) of their lives in menopause or perimenopause.(3)

The United Nations statistical projections demonstrate rapid growth of elderly population in Iran. While the proportion of people with 60 years old age and above in Iran was 5.4% in 1975, it will increase to 10.5% in 2025 and 21.7% in 2050. In fact the total size of population of Iran will fail to double in the next fifty years, but the number of elderly aged 65 years and over will experience about six-fold increase. Thus, it is no longer possible to ignore the commencing aging phenomenon in Iran and therefore, it is vital to anticipate requirements of this age group in Iran to plan appropriate policies to address their growing needs and to support their quality-of-life.(4) It appears that signs and symptoms of menopause are going to numerous, but fortunately, all of them would not aggregate in one individual and some women never experience any of them. Despite that, it is estimated that about 75% of women experience acute symptoms after menopause. These signs are grouped as short- medium- and long-term according to the time they appear. Short-term signs include irregular bleeding, vasomotor signs, myoarthralgia, sexual dysfunction, skin and urinary signs and psychological signs. Long-term signs include osteoporosis and cardiovascular diseases.(5) Vasomotor flushing is one of the most common and the most annoying sign in climacteric women. Among these women, 85% experience it for more than one year and 25–50% for more than five years.(6) These women may complaint about secondary symptoms of vaginal dryness such as dyspareunia and these symptoms can cause decreased libido and sexual dissatisfaction. Women may present with dysuria, urgency and frequency or other signs associated with urinary infection such as discharge, irritation or bleeding.(7) Concurrent with the physical changes occurring in perimenopause, psychological symptoms are also experienced that may disturb patient's life.(3) The most important aid offering by the clinician to these women, is education and awareness on perimenopausal changes and available therapeutic options. The main objective of which is to lengthen the stage at which the individual has optimum physical energy and mental strength for social activity.(2) One of the goals of health services for all of the people in the 21^st^ century is to improve quality-of-life.(8) By WHO definition, quality-of-life is the individual's perception of their status in life according to the cultural and value systems the person lives in, considering their aims, expectations, standards and worries.(9) In menopausal women, quality-of-life usually refers to aspects pertaining to health based on a combination of symptoms without considering physical, emotional or social function.

Therefore, the term quality-of-life in menopause women often simply refers to the climacteric symptoms of flushing, night sweats and vaginal dryness. Definitely, these signs affect the women's quality-of-life; other aspects of personal health, self satisfaction, and mental function are likewise important.(10)

The present study was conducted, addressing the importance of improving health standards and quality-of-life in menopausal women, to evaluate the effect of education on the quality-of-life in this group of people.

## Materials and Methods

This was an experimental study in which the effect of education on the quality-of-life in menopausal women referring to Motahhari clinic (an academic outpatient clinic) in Shiraz was evaluated during 2007–2008. The Medical Research Ethics Committee of Shiraz University of Medical Sciences approved the study. All of participants signed informed consent and they were assured of guarantees of confidentiality and anonymity during and after the study.

From power calculation, by means of a prior pilot study, 31 women in each treatment group with at least 25 completing the study were needed for 95% power to detect at least 5% difference in the quality-of-life.

Patients considered for this study were healthy, premenopause or menopause women.(11) Inclusion criteria were following: 1. having symptoms of moderate to severe hot flashes happening at least once a day; 2. naturally premenopause (regular menstrual cycles during the last three months), perimenopause (3–11 months of amenorrhea or increased menstrual irregularity if still cycling) and postmenopausal (12 or more months of amenorrhea); 3. Not using any kinds of medication and/or hormone replacement therapy 6 months prior to the study (herbal/chemical); 4. not doing physical exercises more than 5 min per day and 20 min per week; 5. to be married; 6. to be aged from 44 to 55 years old. If any of the subjects did not meet even one characteristic of inclusion criteria, she would be drawn off the study and 7. lack of illnesses creating hot flash-like symptoms or impairing quality-of-life.

Scores for quality-of-life in the study and control groups were evaluated and compared in two stages (before educational intervention and three months after educational intervention) both between and within the groups as changes before and after education.

In order to collect data on quality-of-life, we used a questionnaire containing 29 questions about quality-of-life in menopause designed by Hilditch.(12) The quality-of-life questionnaire for menopausal women contains four domains including: vasomotor, psychosocial, physical and sexual aspects.

Of all questions in the original Hilditch questionnaire, we selected and utilized 26 questions in this study (2 questions on vasomotor aspects, 6 questions on psychosocial aspects, 16 questions on physical aspects and 2 questions on sexual aspects). According to the scoring system of original version of Menopause Specific Quality of Life Questionnaire (MENQOL), each question should have been scored by 8 points. However, because of the similarities among some points, two points were omitted in Rotem *et al*. study (13). Using Likert scoring method, each question could be scored by 6 points (1 point: subject had no problem, 2 points: subject had a problem causing mild distress, 3 points: subject had a problem resulting into moderate distress, 4 points: subject had a problem that causes relatively severe distress, 5 points: subject had a problem causing severe distress, and 6 points: subject had a problem causing very severe distress). Hereby, the total scores for vasomotor aspects were from 2 to 12, for psychosocial aspects were from 6 to 36, for physical aspects were from 16 to 96 and for sexual aspects were from 2 to 12. The total score of quality-of-life for each participant could be from 26 (the lowest level) to 156 (the highest level) points. The more the scores decreased, the better the quality-of-life became (13).

Regarding the perception of our participants, their culture and the period of conducting the study, we had to modify the questionnaire in order to make time and cultural adaptation. We omitted three questions from the original MENQOL questionnaire: the item ‘sweating’ was omitted from vasomotor domain, because half of the study was carried out during the end of spring and beginning of summer and the weather was warm-to-hot during the study. If we had put the item ‘sweating’, participants might probably have misreported the daily sweating due to warm weather, not real flashes; because of the different perceptions that Iranian people have, the item ‘avoiding intimacy’ was omitted from sexual domain; it might have been misinterpreted by each of participants and they might have been confused between emotional intimacy, physical intimacy and sexual intimacy; the item ‘dissatisfaction with my personal life’ was skipped from psycho-social domain, because so many factors are influencing the personal life and these factors differ from person to person and community to community. Regarding these changes, we rechecked the validity and reliability of new modified questionnaire. We proceeded to evaluate our modified questionnaire and six-point scoring system. By recruiting a panel of experts to review the test specifications and the selection of items, the content validity of modified questionnaire was approved. The experts were able to review the items and comment on whether the items cover a representative sample of each domain. Moreover, using modified questionnaire, we did a pilot study on 25 menopausal women referring to an outpatient clinic and Cronbach α approved the reliability of questionnaire (0.85).

Participants randomly allocated in two groups as control and study groups in the following way: we listed all of the eligible people and assigned each member of the population a numerical label. Then, using a random table, we pointed a finger on the table in order to choose an arbitrary and random starting point. The one, who possessed that number, was the first participant in study group. Next, we moved across the row of numbers to the very next number to select the first subject in control group. If the next number was less than 62, it was taken as a sample. We continued and assigned every other number to each of the groups until we had two groups that had 31 participants in each of them.

After an initial evaluation and estimation of educational needs, educational intervention was performed weekly, for six consecutive weeks; each section lasted 45–60 min, sittings in the form of eight-person discussion groups. The content of the educational sessions were following: to give information about female genital organs, the definition of menopause and how it happens (the first session); to describe the symptoms and complications of menopause (the second session); to offer some approaches in order to diminish menopausal complications (the third and fourth session); to present training programs on exercise and its effect on the menopausal symptoms (the fifth session); and to describe muscle relaxation techniques and its effects on the severity of menopausal complications (the sixth session). At the end of each session, the summary of the instructed program was delivered to participants and at the end of the whole period of education, a booklet containing all of presented programs accompanied with the CD for relaxation techniques were given to the participants in the study group.

The control group received no education and they had no contact with study personnel (or other participants) beyond recruitment and data collection.

At third month after intervention, entire participants of two groups completed the MENQOL questionnaires.

Data was processed using SPSS version 11.5 for windows. We used paired *t*-test in order to compare the mean scores for diverse criteria (total score for quality-of-life) within groups before and after the study. Independent *t*-test was used in order to compare the mean scores between groups before and after the study. Chi-square test was used for determining the differences among demographic status between groups and the quality-of-life measures in relation to age, the mean age at menopause and level of education. A *P* value less than 0.05 was considered as statistically significant.

## Results

Thirty-one women in each study group completed the study. The mean age of participants was 50.63 ± 2.7. The demographic status of both study groups were evaluated at the baseline (age, education, job status) and there were no statistically significant differences in all baseline parameters between groups (*P* > 0.05) [Table 1].

**Table 1:** The demographic status of participants in both study and control groups^a^

Demographic status groups	Premeno-pause	Perimeno-pause	Postmeno-pause	Housewife	Employed	Elementary school	Middle junior school	High school
Study group	5 (13.6)	6 (21.9)	20 (64.5)	25 (80.64)	6 (19.36)	23 (71.9)	3 (11.3)	5 (15.8)
Control group	5 (13.6	7 (25.1)	19 (61.3)	24 (77.41)	7 (22.59)	22 (70.8)	5 (16.1)	3 (13.1)

a Values are given as number (percent).

After educational intervention, a salient improvement was seen in the mean score for vasomotor symptoms compared to the scores prior to intervention (*P* = 0.004). In the control group, comparing the scores before study, after study showed a statistically significant drop (*P* = 0.004).

The score for psychosocial function in the study group improved after intervention (*P* = 0.001). However, in the control group a significant deterioration was registered (*P* = 0.001).

Three months after intervention, mean scores for physical wellbeing compared to base scores in the study group showed a significant improvement (*P* = 0.001). In the control group, it showed a significant negative growth (*P* = 0.001).

In terms of sexual health, the study group showed a significant improvement (*P* = 0.001). Whereas, control group showed a significant opposite movement (*P* = 0.001).

Three months after education, score for quality-of-life in the study group was improved significantly (*P* = 0.001). Nevertheless, control group showed a significant deterioration in their sense of wellbeing (*P* = 0.001) [Table 2].

**Table 2:** The mean scores for diverse criteria and quality-of-life within groups in both study and control groups

		N	Mean scores before intervention	Mean scores 3 months after intervention	SD	t	*P* value
Vasomotor	S	31	7.5	5.2	2.6	4.7	0.004
	C	31	8.8	6.8	1.3	−3.9	0.004
Psychosocial	S	31	19.6	15	3.2	7.9	0.001
	C	31	20.2	21.1	1.9	−2.8	0.001
Physical	S	31	47	44.4	21.8	5.1	0.001
	C	31	40.3	41.2	1.3	−3.3	0.001
Sexual activity	S	31	7.5	4.7	1.7	8.4	0.001
	C	31	6.3	7.1	1.1	−3.8	0.001
Quality-of-life	S	31	81.7	75.3	6.4	7.6	0.001
	C	31	74.8	75.8	1.4	−3.7	0.001

*N* = number, SD = standard deviation (within-group change), S = study group, C = control group

There was a statistically significant difference between study and control groups, three months after educational intervention, according to the mean score for vasomotor symptoms, psychosocial aspect, physical wellbeing, sexual health and quality-of-life (*P* = 0.001) [Table 3].

**Table 3:** Comparison of the mean scores for diverse criteria and quality-of-life between groups

		N	Mean scores before intervention	Mean scores 3 months after intervention	SD	t	*P* value
Vasomotor	S	31	7.5	5.2	0.53	6	0.001
	C	31	8.8	6.8			
Psychosocial	S	31	19.6	15	0.67	5.7	0.001
	C	31	20.2	21.1			
Physical	S	31	47	44.4	1.4	12.4	0.001
	C	31	40.3	41.2			
Sexual activity	S	31	7.5	4.7	0.37	9.6	0.001
	C	31	6.3	7.1			
Quality-of-life	S	31	81.7	75.3	2.2	13.47	0.001
	C	31	74.8	75.8			

*N* = number SD = standard deviation (follow-up measurement between groups), S = study group C = control group. The flow of participants through each stage of the randomized trial

## Discussion

The goal of the present intervention was improving quality-of-life. As a rule, simple but effective interventions would be beneficial for all kinds of diseases and discomforts.

Setting a random sampling and using self-administered questionnaires that the researcher did not have any role in completing them, we aimed to conduct a bias-free study. Using discussion group and putting participants in touch with the others, women were able to talk with each other about their experiences.

The finding of the study showed no significant difference between the study and control groups in terms of demographic characteristics (age, level of education and the mean age at menopause was not different between groups). Many studies have shown that differences in demographic characteristics could affect the women's quality of lives. According to Peter Chedraui *et al*. study in Ecuador,(13) the quality-of-life in climacteric women was related to age, and the quality-of-life scores were lower in younger women (given the higher scores in vasomotor aspect). As can be seen, most of the women in our study had elementary school education and they presented dissatisfactory quality of lives prior to study. It was in accordance with the findings of the other studies. In Blumet study this same relationship was noted. In addition, quality-of-life scores were lower in individuals with lower levels of education.(14)

Due to the finding in our study that the majority of subjects had a lower level of education and a lack of proper sources of information (i.e. having access to books, magazines and/or any educational programs), the need for planning and implementation of an educational program becomes more apparent.

Regarding the role of muscle relaxation presented in this study, while evaluating the vasomotor aspect, it can be seen that the scores gained in the study group, three months after the intervention, were better than those before the intervention (*P* = 0.004) and the difference between post-intervention scores of the study group and those of the control group was significant (*P* = 0.001). Therefore, it can be said that the intervention led to an improvement in vasomotor symptoms of the study group. Our findings are similar to those of Booth-Laforce *et al*.,(15) who reported a decrease in hot flushes following the practice of Yoga. Yoga, as we know, is a proper method to induce relaxation and decrease distress.

Our finding showed a significant improvement in psychosocial wellbeing in the study group, three months after intervention (*P* = 0.001); while the control group had a significant deterioration. Likewise the comparison of mean score, in the same aspect, showed a significant difference between the study and control groups after intervention (0.001). Therefore, the applied intervention led to improvement of psychosocial status in participants. This finding is similar to the result of Elavsky *et al*.(16) study, which noted an improvement following physical exercises in menopause women. The study group registered a significant physical health status improvement 3 months after intervention. This result is similar to that of Rotem *et al*.(17) who reported improvement in physical health status following education in menopause women.

Following betterment in hot flashes, the study group showed a significant improvement in sexual health, which their changes were significantly better than that of the control group. Lobo *et al*.(18) pointed out that the sexual aspect of a menopause woman's life could be recovered indirectly following the improvement of hot flashes. Furthermore, using similar training approaches, Osinowo(19) in Nigeria expressed that after informing women on menopause, an improvement of self-perception marital satisfaction and sexual activity was seen.

Quality-of-life improved significantly in the study group following intervention while the controls significantly regressed in the same. The mean scores in the study group, in addition, were significantly better following their education. This finding confirms that of Keefer *et al*.(20) who reported an improvement in the quality-of-life following education on menopause symptoms and coping skills in the study group while the controls, due to progressive nature of climacteric complications, registered some worsening.

The finding of this study asserted that the four aspects of quality-of-life well improved after educational intervention, and the education can cause an improvement in the quality-of-life by decreasing the problems of menopause stage and lowering their intensity,. Therefore, the urgency of need to planning and implementing an appropriate educational program is emphasized in order to promote the quality-of-life in this group of people.

## Conclusions

This study was conducted to evaluate the effect of education on the quality-of-life and the improvement of health standards in menopausal women. Our findings showed that an appropriate training to menopausal women can improve their quality-of-life and promote their health.
